# Ungewöhnliche Manifestationsform einer allergischen bronchopulmonalen Aspergillose während des COVID-19-Lockdowns. Ein Fallbericht

**DOI:** 10.1159/000516041

**Published:** 2021-03-26

**Authors:** Daniela Savi, Giada Valente, Alessandra Iacovelli, Federica Olmati, Mario Bezzi, Paolo Palange

**Affiliations:** 1 ^a^Fakultät für Öffentliche Gesundheit und Infektionskrankheiten, «Sapienza» Universität Rom, Rom, Italien; 2 ^b^Fakultät für Radiologie, Onkologie und Pathologie, «Sapienza» Universität Rom, Rom, Italien

**Keywords:** Coronavirus-Krankheit 2019 (COVID-19), Allergische bronchopulmonale Aspergillose (ABPA), *Aspergillus*-assoziierte Erkrankungen des Respirationstrakts

## Abstract

**Hintergrund:**

Die Lockdown-Phasen während der anhaltenden Coronavirus-Pandemie mit der Erkrankung COVID-19 haben die Art und Weise verändert, wie Personen und Gemeinschaften leben, arbeiten und interagieren.

**Fallvorstellung:**

Der vorliegende Fallbericht beschreibt eine ungewöhnliche, aber bedeutende Manifestationsform der allergischen bronchopulmonalen Aspergillose (ABPA) bei einem zuvor gesunden Mann, der zu Beginn des landesweiten Lockdowns in Italien während der COVID-19-Pandemie beschloss, in den Keller seines Hauses zu ziehen. Da eine hochauflösende Computertomographie (HRCT) des Thorax bei Aufnahme des Patienten diffuse miliäre Noduli zeigte, bestand initial der Verdacht auf eine Miliartuberkulose. Weitergehende Untersuchungen führten allerdings zur Diagnose einer ungewöhnlichen Manifestationsform der ABPA.

**Schlussfolgerungen:**

Der vorliegende Fall unterstreicht die Bedeutung einer ungebrochenen Aufmerksamkeit in Bezug auf Aspergillus-assoziierte Erkrankungen des Respirationstrakts während der COVID-19-Pandemie, insbesondere aufgrund dessen, dass Änderungen der Lebensführung im Zusammenhang mit häuslicher Isolation mit dem erhöhten Risiko für eine Exposition gegenüber Schimmelpilzsporen in einigen Wohnräumen verbunden sind.

## Hintergrund

Neben den schädlichen Auswirkungen auf die Lunge und andere Organe kann die Coronavirus-Krankheit 2019 (COVID-19) anerkanntermaßen direkte und indirekte psychologische und soziale Folgen mit Wirkung auf die mentale Gesundheit haben [1]. So können Zeiten der Quarantäne zu einer depressiven Symptomatik und Schlafstörungen mit nachfolgenden Verhaltensänderungen in der Bevölkerung führen [1]. Der vorliegende Fallbericht beschreibt eine ungewöhnliche, aber bedeutende Manifestationsform der allergischen bronchopulmonalen Aspergillose (ABPA) bei einem Mann, der zu Beginn des landesweiten Lockdowns in Italien während der COVID-19-Pandemie beschloss, in den Keller seines Hauses zu ziehen.

## Fallvorstellung

**Abb. 1:**
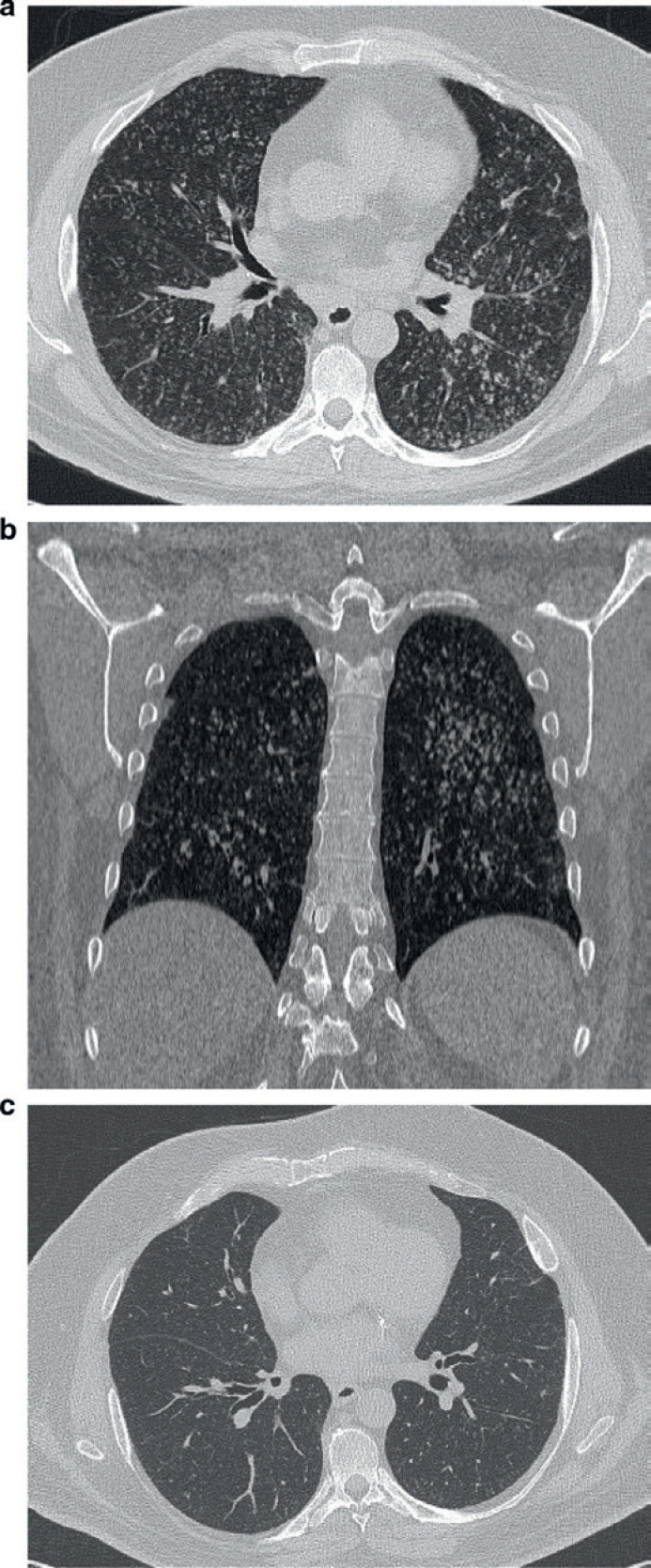
HRCT. (**a**) Das axiale Thorax-CT zeigt multiple kleine, unscharfe noduläre Verdichtungen mit diffuser Verteilung über beide Lungenfelder. (**b**) Das Thorax-CT in der Frontalebene zeigt ein *Tree-in-Bud*-Muster im linken unteren Lungenfeld. (**c**) Das axiale Thorax-CT drei Monate nach der stationären Aufnahme zeigt eine Reduktion der multiplen nodulären Verdichtungen.

Während der COVID-19-Pandemie stellte sich im April 2020 ein 55-jähriger Mann europäischer Herkunft mit Fieber bis zu 38,6°C, Husten und Kurzatmigkeit in der Notaufnahme unserer Einrichtung vor. Seine Anamnese umfasste ischämische Herzkrankheit, Diabetes, arterielle Hypertonie, schwere Adipositas sowie Asthma, und er war Raucher. Aufgrund der respiratorischen Insuffizienz wurde sofort eine Sauerstofftherapie über eine Venturi-Maske mit einer Konzentration von 35% eingeleitet. Bei der Auskultation des Brustkorbs waren beidseitig Rasselgeräusche zu hören; die körperliche Untersuchung ergab keine weiteren relevanten Befunde. Das Blutchemie-Profil (Tab 1) zeigte eine hohe Leukozytenzahl sowie hohe Konzentrationen für C-reaktives Protein (CRP) und D-Dimer (letztgenannter Wert 1652 mcg/l; Normalwert: 0–550 mcg/l). Aufgrund der anhaltenden Pandemie und in Anbetracht seiner Symptomatik wurde der Patient zunächst als COVID-19-Verdachtsfall behandelt, obwohl zwei aufeinander folgende Nasen-Rachen-Abstriche negativ für SARS-CoV-2 waren. Vor und nach einer intravenösen Injektion jodhaltigen Kontrastmittels wurde eine hochauflösende Computertomographie (high-resolution computed tomography, HRCT) des Thorax durchgeführt. Die Untersuchung zeigte multiple kleine, unscharfe noduläre Verdichtungen mit diffuser Verteilung über beide Lungenfelder und ohne spezifische Lappenpräferenz (Abb 1a, b). Die Noduli waren zentrilobulär lokalisiert und zeigten ein lineares Verzweigungsmuster sowie ein *Tree-in-Bud*-Muster, hauptsächlich erkennbar im linken unteren Lungenfeld (Abb 1b). Im CT zeigten sich ebenfalls leicht vergrößerte mediastinale Lymphknoten, ohne Anzeichen für eine Nekrose, vermutlich reaktiver/entzündlicher Natur. Der Einschätzung zufolge ließ der HRCT-Befund nicht auf eine COVID-19-Pneumonie schließen.

Es wurde die Verdachtsdiagnose einer Miliartuberkulose bzw. anderen diffusen infektiösen/entzündlichen Erkrankung gestellt, und der Patient wurde an unsere Abteilung für Atemwegserkrankungen zu weiteren Untersuchungen überwiesen. Die Anamnese ergab keine Hämoptyse, keine Brustschmerzen und keinen Gewichtsverlust. Der Patient berichtete, er habe während der Pandemie beschlossen, in das feuchte Kellergeschoss seines Hauses zu ziehen und sich von seiner Familie zu isolieren. Er habe dort regelmäßig mehrere Stunden pro Tag gearbeitet. Dies ereignete sich sechs Wochen vor Auftreten der Symptome und war die einzige wesentliche Veränderung in seinem Alltag.

Drei Sputumproben waren negativ für säurefeste Stäbchen, und auch ein Mendel-Mantoux- sowie ein QuantiFERON-Test erbrachten negative Ergebnisse. Daher wurde eine Tuberkulose ausgeschlossen. Darüber hinaus wurde eine Pneumonie infolge einer Infektion mit *Legionellasp.*, *Mycoplasmasp*. oder *Chlamydiasp.* ebenso ausgeschlossen wie eine HIV-Infektion und Autoimmunerkrankung. Ein sekundäres Neoplasma der Lunge stellte eine weitere Verdachtsdiagnose dar, aber ein Ganzkörper-CT ergab keinerlei maligne Veränderungen. Es wurde eine empirische Antibiotikatherapie mit Ceftriaxon 2 g/Tag und Azithromycin 500 mg/einmal täglich über sieben Tage eingeleitet. Drei Wochen nach der stationären Aufnahme wurde bei dem Patienten ein erneutes, nicht kontrastmittelverstärktes Thorax-CT durchgeführt. Diese zeigte ein Persistieren des oben beschriebenen Musters. Tests auf antinukleäre und antineutrophile Zytoplasma-Antikörper waren negativ. Der klinische Zustand des Patienten verbesserte sich geringfügig: Er war fieberfrei, und die Konzentration der Sauerstofftherapie über die Venturi-Maske wurde daraufhin umgehend auf 24% reduziert. Das Blutchemie-Profil zeigte eine normale Leukozytenzahl und einen verminderten CRP-Wert (Tab 1). Der Patient unterzog sich einer Bronchoskopie, und die bronchiale Lavageflüssigkeit (BLF) ergab einen Galactomannan-Spiegel von 3,7 (Obergrenze des Normbereichs: 0,5). Das Gesamt-Immunglobulin E (IgE) im Serum war erhöht (1016 IE ml^1^; Normalwert: <100 IE ml^1^), obwohl das *A. fumigatus*-spezifische IgE negativ war. Das *A. fumigatus*-spezifische IgG war ebenfalls erhöht (Tab 1). Eine Spirometrie ergab eine restriktive Ventilationsstörung, die nach der Gabe von Salbutamol reversibel war, was die Asthma-Diagnose bestätigte. Die forcierte Vitalkapazität (FVC) betrug 3,22 l (70% des Sollwerts); das forcierte exspiratorische Volumen in 1 Sekunde (FEV_1_) betrug 2,23 l (61% des Sollwerts) und 3,00 l (+34,4%) nach Bronchodilatation. Die BLF-Kultur war positiv für *Pseudomonas aeruginosa* rugosa und *Achromobacter xylosoxidans*. Abschließend waren folgende diagnostischen Kriterien für eine ABPA erfüllt: (1) Prädisponierende Erkrankung wie Asthma bronchiale; (2) erhöhtes Gesamt-IgE (>1000 IE ml^1^); (3) IgG-Antikörper im Serum gegen *A. fumigatus*; (4) radiologisch nachgewiesene Verdichtungen in der Lunge, die mit einer ABPA konsistent sind. Ein Hauttest auf eine Überempfindlichkeitsreaktion vom Soforttyp gegenüber *Aspergillus*-Antigen wurde nicht durchgeführt, da der Patient systemisch mit Antihistaminika behandelt wurde. Es wurde eine Therapie mit Prednisolon (0,5 mg/kg/Tag) über vier Wochen eingeleitet. Um die Antigenlast zu verringern, wurde außerdem eine Behandlung mit oralem Isavuconazol mit einer Aufsättigungsdosis von 200 mg dreimal täglich in den ersten drei Tagen und anschließend 200 mg einmal täglich über acht Wochen eingeleitet. Der Patient wurde zwei Monate nach der stationären Aufnahme in einem verbesserten klinischen Zustand, ohne Sauerstofftherapie und mit einem reduzierten Gesamt-IgE im Serum von 306 IE ml^1^ entlassen. Er setzte die Steroidbehandlung fort, deren Dosis alle zwei Wochen stufenweise reduziert wurde. Drei Monate nach der stationären Aufnahme kam der Patient in unsere Ambulanz und berichtete lediglich leichte Dyspnoe bei körperlicher Anstrengung. Das Thorax-HRCT zeigte eine deutliche Verringerung der multiplen nodulären Verdichtungen (Abb 1c), und das Lungenmuster hatte sich fast wieder normalisiert. Das Gesamt-IgE betrug 335 IE ml^1^. Die Prednisolon-Dosis wurde ebenfalls reduziert, und die Behandlung wird zum Zeitpunkt dieser Fallvorstellung mit einer Dosis von 10 mg täglich weiter fortgeführt.

## Diskussion und Schlussfolgerungen

ABPA ist eine Überempfindlichkeitserkrankung der Lunge, verursacht durch eine (IgE-vermittelte) Immunantwort auf Antigene der *Aspergillus*-Spezies, insbesondere *A. fumigatus*. Die Pathogenese der ABPA ist komplex, wobei sowohl genetische als auch immunologische Faktoren eine Rolle spielen. Sie tritt bei erwachsenen Asthma-Patienten sowie in allen Altersgruppen von Patienten mit zystischer Fibrose auf [2]. Als Hauptursache gilt eine Exposition gegenüber hohen Sporenkonzentrationen in feuchten und nassen Gebäuden und mancherorts sogar im Freien. Die ABPA manifestiert sich mit schlecht beherrschbarem Asthma, rezidivierenden Lungeninfiltraten und Bronchiektase [2]. Die Erkrankung ist in vielen Ländern unterdiagnostiziert, und bis zu zwei Drittel der Fälle werden als Lungentuberkulose fehldiagnostiziert [3]. Der HRCT-Befund bei ABPA umfasst Bronchiektasien, Sekretstau und zentrilobuläre Noduli mit einem *Tree-in-Bud*-Muster. Eine Beteiligung der Pleura ist eher selten und manifestiert sich mit Pleuraerguss und Pleuraverdickung. Es kann zu fibrotischen Veränderungen bis hin zur Fibrose im Endstadium kommen [3]. Nach einer ABPA-Diagnose ist es wichtig, die Entwicklung von Bronchiektasien − einer Manifestationsform dauerhafter Lungenschäden bei ABPA − zu verhindern bzw. zu verzögern.

Die Diagnose stützt sich auf die Kombination aus klinischem Bild und nachgewiesener Überempfindlichkeitsreaktion auf *Aspergillus*. Da kein Kriterium für sich genommen pathognomonisch ist, wurden Leitlinien erarbeitet, um die Kombination klinischer, radiologischer und immunologischer Merkmale, die zur Diagnose ABPA führen, leichter bestimmen zu können [4].

Es gibt fünf Stadien der ABPA (Akutstadium, Remission, Exazerbation, kortikoidpflichtiges Asthma, fibrotische Veränderungen des Lungengewebes), wobei in keinem dieser Stadien bislang das miliare Muster beschrieben ist [5]. Die radiologischen Aufnahmen bei ABPA unterscheiden sich je nach Krankheitsstadium. So können in der Akutphase homogene Infiltrate, Schleimpfropfbildung, lobäre Konsolidierung, ein *Tree-in-Bud*-Muster und Bronchiektasien vorliegen [6]. Im HRCT zeigen sich bei Patienten mit ABPA in der Regel zentrale Bronchiektasien.

Der vorliegende Fall ist sowohl aus radiologischer als auch aus soziologischer Sicht aufgrund des irreführenden klinischen Bildes der ABPA und der Rolle der häuslichen Isolation aufgrund des COVID-19-Lockdowns interessant. Laut einer Literaturrecherche ist dies der vierte Fall einer ABPA, die sich in Form von zufällig verteilten, unscharfen Noduli mit einer Verteilung über beide Lungenfelder manifestiert [7, 8, 9]. Die ungewöhnlichen radiologischen Befunde, d.h. ein miliares Muster und zentrilobuläre Noduli, sind in vielen Fällen der Grund dafür, dass eine ABPA fälschlich als Lungentuberkulose diagnostiziert wird.

Darüber hinaus belegt dieser Fall die sozialen Auswirkungen der Isolation aufgrund des Lockdowns während der COVID-19-Pandemie. Als eine Folge der Quarantäne entwickelte unser Patient neue Verhaltensweisen in seinem Alltag, u.a. verstärkt IT-gestütztes Arbeiten (*smart working*) und ein verstärktes Bedürfnis nach Privatsphäre. Bei einem entsprechend veranlagten Wirtsorganismus wie im Falle unseres Patienten könnten längere Aufenthalte in feuchtkalten Räumen zu einer wiederholten Inhalation von *Aspergillus*-Sporen und in der Folge einer Besiedlung der Atemwege führen, die eine allergische Reaktion hervorruft. Im vorliegenden Fall war die besondere häusliche Umgebung, die zur Exposition gegenüber *A. fumigatus* führte, ausschlaggebend.

Die andauernde COVID-19-Pandemie mahnt uns nachdrücklich, dass Lockdown-Phasen die Art und Weise verändern, wie Personen und Gemeinschaften leben, arbeiten und interagieren, und unterstreicht die Notwendigkeit einer adäquaten, angenehmen Lebens- und Arbeitsumgebung − sowohl im Haus als auch im Freien. Eine aktuelle Studie hebt die Bedeutung möglichst flexibel nutzbarer Wohnräume (Räume im Erdgeschoss, Kellerräume, freie Räume) hervor, die einfach angepasst werden können, um beispielsweise dort vorübergehend einen ruhigen Arbeitsplatz einzurichten und genügend Abstand einzuhalten (*social distancing*) [10].

Insgesamt kann die Diagnose einer ABPA schwierig sein, da nicht in allen Fällen sämtliche Kriterien erfüllt sind und die Krankheit unterschiedliche radiologische Manifestationsformen aufweisen kann. Wie eine Durchsicht der Literatur zeigt, ist ein CT-Befund, der lediglich zufällig verteilte Noduli und ein teilweises *Tree-in-Bud*-Muster zeigt, für eine ABPA nicht charakteristisch. Dies legt nahe, dass die Beschreibung des akuten Krankheitsstadiums um dieses Muster ergänzt werden sollte. Eine frühe Diagnose und Behandlung solcher Fälle von ABPA werden dazu beitragen, die Entwicklung einer Lungenfibrose im Endstadium zu verhindern. Der vorliegende Fall unterstreicht die Bedeutung eines hohen Sensibilisierungsgrads für mögliche *Aspergillus*-assoziierte Erkrankungen des Respirationstrakts während der COVID-19-Pandemie, insbesondere aufgrund dessen, dass Änderungen der Lebensführung im Zusammenhang mit häuslicher Isolation das Risiko für eine Exposition gegenüber Schimmelpilzsporen in einigen Wohnräumen erhöhen können.

## Disclosure Statement

Die Autoren erklären, dass keine Interessenskonflikte bestehen.

## Lizenzangabe

Daniela Savi, Giada Valente, Alessandra Iacovelli, Federica Olmati, Mario Bezzi, Paolo Palange: Uncommon presentation of allergic bronchopulmonary aspergillosis during the COVID-19 lockdown: a case report. BMC Pulm Med. 2020; 20(1): 325 (DOI 10.1186/s12890–020–01373–7). ^©^ 2020 Die Autoren (Übersetzung; Abkürzungen, Danksagung, Autorenbeiträge, Finanzierung der Studie, Verfügbarkeit der Daten und Materialien, Genehmigung durch die Ethikkommission und Einwilligung in die Teilnahme und Zustimmung zur Veröffentlichung gekürzt), lizensiert unter CC BY 4.0 (https://creativecommons.org/licenses/by/4.0/deed.de).

## Figures and Tables

**Tab. 1:** Ergebnisse der Blutuntersuchung bei Aufnahme

Tag der Aufnahme	Leukozytenzahl (× 10^9^/l)	Neutrophile (× 10^9^/l)	Lymphozyten (× 10^9^/l)	Eosinophile (× 10^9^/l)	CRP (mg/dl)	D-Dimer (mcg/l)	Gesamt IgE (IE ml^−1^)	IgE *A. fumigatus* (kU/l)	IgG *A. fumigatus* (mg/l)
Tag 1	12,08	10,47	0,72	0,20	31,15	1652			
Tag 21	5,97	4,48	0,94	0,40	0,9	1257	1016	0,09	52,7

*CRP:* C-reaktives Protein, *IgE:* Immunglobulin E im Serum, *IgG:* Immunglobulin G im Serum
